# Contrast in utilization of maternal and child health services between Himalayan region and rest of India: Evidence from National Family Health Survey (2015–16)

**DOI:** 10.1186/s12884-021-04081-0

**Published:** 2021-09-05

**Authors:** Akif Mustafa, Chander Shekhar

**Affiliations:** 1 Research Fellow, International Institute for Population Sciences (IIPS), Mumbai, 400088 India; 2 Department of Fertility Studies, International Institute for Population Sciences (IIPS), Mumbai, India

## Abstract

**Background:**

Maternal and child health services, like antenatal care, skilled birth attendance and postnatal care, are crucial to improve maternal and neonatal health outcomes. Numerous studies have been conducted on the distribution of utilization of maternal and child healthcare (MCH) services in India with respect to socioeconomic and demographic characteristics. But no study has analyzed the utilization of MCH services with a focus on the topography of a given region (hilly/plain). The present study analyzes the utilization of MCH services in the hilly-Himalayan region of India in comparison to the rest of the country.

**Methods:**

Data from India’s National Family Health Survey-4 (2015–16), on 190,898 women, was utilized for analysis in the present study. The association between the utilization of MCH services and the topography of the region of residence (hilly/plain) was analyzed by calculating adjusted odds ratios (AOR) with 95% confidence interval (95%CI) and predicted probabilities using a two-level random intercept logistic regression model.

**Results:**

It was found that the utilization of MCH services was significantly lower in the hilly regions compared to the plain regions. Women living in hilly areas (AOR: 0.42, 95%CI: 0.39–0.45) had 58% lower odds of receiving skilled birth attendance (SBA) than those living in plain areas. Similarly, the odds of receiving PNC, ANC, and full immunization were also lower in the hilly regions compared to the plain regions. The utilization of MCH services was alarmingly low in the rural-hilly regions. The odds of receiving two tetanus injections before birth were 71% lower for women in the rural-hilly areas (AOR: 0.39, 95% CI: 0.36–43) than those in the rural-plain areas. Predicted probabilities also showed that women in the hilly regions were less likely to receive MCH services compared to their counterparts in the plain regions.

**Conclusion:**

Except for the consumption of Iron Folic Acid (IFA) and the utilization of AWC services/ICDS (Integrated Child and Development Services), all other MCH services were significantly underutilized in the hilly regions compared to the plain regions. This calls for the attention of and concentrated efforts by policy makers and stakeholders, with a special focus on the rural-hilly regions. We firmly believe that the results of the present study have important policy implications.

**Supplementary Information:**

The online version contains supplementary material available at 10.1186/s12884-021-04081-0.

## Introduction

A major proportion of the child and maternal mortalities occur during child birth and the postnatal period, with most deaths occurring in the first 24 h of birth [1–3]. Although in the past few decades, there has been a significant improvement in maternal and child health, a lot more needs to be done, especially in the low- and middle-income countries. In 2017, approximately 2,95,000 women died around the world, 94% of them being from the low- and middle-income countries (LMICs), due to complications related to pregnancy and child birth [4]. WHO has stated that in the year 2017, 4.1 million infants lost their lives before their first birthday and that 808 women died daily due to complications related to pregnancy and child birth [5, 6]. In 2019, Unicef reported that 2.4 million children died globally in the first month of their lives and that the major proportion of these deaths came from Sub-Saharan African and South-Asian countries [7]. Approximately 26% of the Indian population is composed of women of reproductive age [8]. Infant mortality in India declined significantly to 32 infant deaths per 1000 live births in 2019 from 129 infant deaths per 1000 live births in 1971 [9]. In spite of this, the highest number of neonatal mortalities still occur in India. Until 2015, one-fifth of the global under-five deaths were contributed by India [10]. Maternal mortality ratio currently stands at 113 in India [11].

Maternal and child healthcare (MCH) services play a crucial role in minimizing the risks related to pregnancy and childbirth. Although maternal and child mortality rates cannot be curbed by a single intervention, previous studies show that providing skilled assistance at the time of delivery, post- neo- and ante-natal care, and putting a strong healthcare system in place can significantly help in reducing maternal and child mortalities [12–14]. Antenatal care (ANC) has great significance in maternal and child healthcare. Apart from helping in detecting the risks and complications related to pregnancy, ANC also aids in health education, tetanus immunization, vaccination, and other treatments [15, 16]. Advocating “skilled care at every birth”, WHO states that quality maternity care services can save the lives of women and newborns [13]. Postnatal care can help in identifying post-delivery risks and is instrumental in preventing child and maternal mortality [14]. In India, utilization of postnatal care increased to 65% in 2015–16 from 37% in 2005–06 [17]. According to the National Family Health Survey (NFHS-4), the proportion of women receiving ANC increased to 84% in 2015–16 from 80% in 2005–06 [17]. During the same period, the percentage of institutional deliveries doubled from 39 to 79% [17]. It is true that in the past few years, India has made significant improvements in the utilization of MCH services, but large socioeconomic and geographic inequalities still persist in the country.

People living in the hilly regions face many issues related to poor accessibility and unmatched supply and demand. Lack of employment opportunities, poor connectivity, water scarcity, and lack of healthcare services are some of the major problems that people living in the hilly regions face. A 2019 report found that in Uttarakhand (a hilly state of India), 8% of the residents migrated from the rural areas to urban areas due to lack of medical facilities [18]. There were even cases where women delivered their babies on roads or in other unsafe conditions [18]. Numerous factors affect the utilization of MCH services like distance to health facility, road connectivity, travel time to health facility, economic status, quality of facilities at health center, mass media exposure, mode of delivery, contact with health workers during pregnancy, among others [19–21]. However, accessibility to healthcare facilities has been found to be one of the most consistent determinants of healthcare service utilization [22–24]. The Rural Health Statistics (RHS, 2019), India, report shows that most of the hilly states, like Arunachal Pradesh, Himachal Pradesh, Nagaland, Sikkim, and Uttarakhand, have enough number of Primary Health Centers (PHCs) and Community Health centers (CHCs) [25]. The report also shows that in these hilly states, the population load on health facilities (population in the catchment area of per PHC/CHC) is significantly lower compared with other states like Bihar, Haryana, Jharkhand and Punjab. This clearly shows that these hilly states have enough health facilities. Nevertheless, the NFHS-4 data shows that health care utilization is significantly low in these states. This phenomenon of low maternal and child healthcare utilization even in the presence of adequate health facilities indicates existence of problems associated with accessibility to health facilities. Distance of health facility from residence is a crucial predictor of healthcare utilization. A previous study shows that the average radial distance covered by a PHC in hilly states, like Mizoram (10.7 km) and Meghalaya (8.05 km), is significantly higher than the national average (6.44), which indicates that the average distance to health facilities in these states is higher compared to the rest of India [26]. Apart from distance, the complex terrain of these states – which is not very favorable for transportation activities and takes a lot of time to navigate – makes delivery and utilization of healthcare services more difficult.

Numerous studies have analyzed the socio-demographic, geographic and economic disparities in the utilization of MCH services in India [27–30]. But we found no study that has exclusively analyzed the state of MCH utilization in the hilly-mountainous region of India. Knowing the degree of paucity and disparity in the utilization of various healthcare services will provide insights into the status of universal health coverage in India and help policymakers identify potential intervention areas. Therefore, in the present study we have tried to analyze the MCH utilization in the hilly Himalayan region of India and compare it with the utilization in the plain areas while keeping the urban-rural dichotomy in mind. The specific objectives of the study are:

1. To assess and compare the utilization of MCH services in the Himalayan region (hilly) and the rest of India (predominantly plain).
2. To examine the disparity in the utilization of MCH cervices in the two geographically divergent regions of India.

## Methodology

### Data source

Data from India’s National Family Health Survey (NFHS-4) was utilized for analysis in the present study. NFHS is the Indian version of the Demographic Health Survey (DHS). NFHS-4 was conducted in 2015–16 under the stewardship of the Ministry of Health and Family Welfare (MoHFW), Government of India, and coordinated by the International Institute for Population Sciences (IIPS), Mumbai [17]. NFHS is a large-scale cross-sectional survey that provides reliable data on a range of topics like fertility, family planning, maternal and child health, domestic violence, sanitation and hygiene, morbidity, nutrition, household amenities and women empowerment, and domestic violence. For data collection, a two-stage stratified random sampling design was adopted in NFHS-4. The survey covered all 29 states, 7 union territories, and all 640 districts as per census 2011. In rural areas, villages, while in urban areas, ‘Census Enumeration Blocks’ were chosen as PSUs. Detailed information about the sampling design, the data collected, and the instruments used can be accessed from the NFHS-4 report [17].

### Sample size

NFHS-4 collected data from 699,686 women aged 15–49 chosen from 28,583 primary sampling units (PSUs), with a non-response rate of 3%. NFHS-4 provides comprehensive data related to live births that happened 5 years preceding the survey. For the present study, information pertaining to only the last live birth (*n* = 190,898) was utilized. Due to the missing values, the sample size was slightly different for different outcome variables. Variables on ANC, tetanus injection, and iron folic consumption had missing values due to recall lapse. For example, 1854 women were unable to recall the number of times they went for ANC, resulting in 1854 missing cases for the ANC variable. Some children may have died in infancy or before becoming eligible for full immunization, which is the reason why the variables of full immunization and ‘Child received benefits from Anganwadi center (AWC)/ICDS’ had some missing cases.

**Table:** 

Outcome Variable	Sample Size	Missing Cases
Institutional delivery	190,337	337
Skilled birth attendance	190,898	0
Post-natal care	190,898	0
At least 4 ante-natal care visits	189,044	1854
Full immunization	184,304	6594
At least two tetanus injections before birth	189,566	1332
Given/took iron folic tablet/syrup for at least 100 days during pregnancy	187,578	3320
Child received benefits from AWC/ICDS in last 12 months	185,101	5797
Received benefits from AWC/ICDS during pregnancy	190,804	94
Received financial assistance for delivery cost	190,337	561
Received financial assistance under JSY to cover delivery cost	190,337	561

### Outcome variables

NFHS provides information on a range of subjects related of MCH services. The present study used 11 variables pertaining to the utilization of MCH services for the analysis. The description of the outcome variables is as follows:

- **Skilled Birth Attendance (SBA):** SBA is defined as a delivery conducted in either in a medical institution or at home assisted by a skilled attendant (doctor/nurse/Lady Health Visitor/Auxiliary Nurse Midwife) [31]. The variable was converted into a binary variable (0 = No skilled attendance at delivery; 1 = skilled attendance at delivery).
- **Post-natal care (PNC):** It is defined as receiving ‘post-natal care/health check-up’ from a health facility or at home within 48 h of delivery for last live birth. The variable was converted into a binary variable (0 = didn’t receive PNC; 1 = received PNC).
- **Ante-natal care (ANC):** ANC is defined as at least 4 ante-natal visits for a pregnant woman, as per the Government of India’s guidelines, to minimize pregnancy-related risks [32]. Data on the number of ANC visit during pregnancy was available in NFHS-4. The variable was converted into a binary variable (0 = less than 4 ANC visits; 1 = at least 4 ANC visits).
- **Full immunization:** This is defined as having one dose of BCG vaccine, three injections against DPT, three doses of polio vaccine, and one vaccine against measles [17]. The variable was converted into a binary variable (0 = received partial or no immunization; 1 = received full immunization).
- **No immunization:** Children who did not get any dose of BCG, polio, and DPT were categorized as having received no immunization. The variable was converted into a binary variable (0 = received partial or full immunization; 1 = received no immunization).
- **At least two tetanus injections before birth:** WHO recommends at least two tetanus injections during pregnancy to avoid the risk of tetanus infection [33]. The respondents were asked about the number of tetanus injections they had received before delivery. The variable was converted into a binary variable (0 = received less than two tetanus injections before birth; 1 = received at least two tetanus injections before birth)
- **Consumed Iron Folic Acid (IFA) tablet/syrup for at least 100 days during pregnancy:** The National Health Mission (Govt. of India) recommends taking iron folate tablets or syrup for at least 100 days during pregnancy to avoid anemia [34, 35]. NFHS-4 provided data on iron folate consumption by mothers during pregnancy. The variable was converted into a binary variable (0 = did not consume iron folate/ consumed iron folate for less than 100 days; 1 = consumed iron folate for at least 100 days)
- **Child received benefits from AWC/ICDS in last 12 months:** Anganwadi centers and Integrated Child Development Services (ICDS) are established to improve health, nutrition, and education of children. Its beneficiaries include children up to age 6 and pregnant and lactating women. In NFHS-4, respondents were asked whether their newborn children had received benefits from AWC or ICDS scheme during 12 months prior to the survey. The variable was binary in nature (0 = No; 1 = Yes).
- **Received benefits from AWC/ICDS during pregnancy:** The variable was binary in nature (0 = No; 1 = Yes).
- **Received financial assistance for delivery cost:** The Government of India aims to provide financial assistance to pregnant and lactating women to cover delivery and other related costs through various schemes. A question was asked to the mothers as to whether they had got any financial assistance to cover delivery costs or not. The variable was binary in nature (0 = No; 1 = Yes).
- **Received financial assistance under the Janani Suraksha Yojana (JSY) for delivery cost:** JSY is a centrally sponsored scheme of India under which cash assistance is provided to mothers for delivery and post-delivery care [36]. The scheme mainly focuses on pregnant women in rural areas. The variable was binary in nature (0 = No; 1 = Yes).

### Exposure variable

The exposure variable of interest was ‘type of region of residence (plain or hilly)’. The states and UTs were divided into two categories: hilly states and plain states. All the states lying in the Himalayan range were categorized as hilly. These states are: Jammu & Kashmir (erstwhile), Himachal Pradesh, Uttarakhand, Sikkim, Arunachal Pradesh, Nagaland, Meghalaya, and Mizoram. Other states like Maharashtra, Madhya Pradesh, and Kerala also have some hilly landscape, but the proportion of hilly area is extremely low. Himalayan states have a significantly higher ecological complexity than hilly areas of the other states. Most of the population in the Himalayan states lives in a knotty hilly terrain because these states have a negligible amount of plain region to live in. Although states like Maharashtra and Madhya Pradesh also have hilly regions, most of those regions are scarcely or unpopulated because the plain region has enough accommodation for the states’ population. This is why, except the Himalayan region states, we did not consider any other state as a hilly state. The present study, therefore, shows disparity in the utilization of MCH services between the hilly states of the Himalayan range (only) and the rest of the country. On the whole, 25,712 respondents were from the hilly Himalayan region, whereas 165,186 were from the plains.

**Table:** 

Type of Area	Sample Size
Hilly	25,712
Plain	165,186

### Control variables

In literature, a range of variables have been found to affect the utilization of child and maternal healthcare services in India [19–21, 37]. In keeping with the literature and the availability of the data, in the multivariate analysis, the association between the outcome and the exposure variables was controlled for the following variables: age of respondent (15–24, 25–34,35–49); age of husband; child marriage (0 = No, 1 = Yes; those married at age 18 or after were categorized as ‘0’, and those married before age 18 years were categorized as ‘1’); place of residence (urban/rural); wealth quintile (poorest, poorer, middle, richer, richest); respondent’s educational attainment (no education, primary, secondary, higher); religion (Hindu, Muslim, Other); Caste (Scheduled Castes (SCs), Scheduled Tribes (STs), Other Backward Class (OBC), Others); sex of head of household (male, female); parity of respondent (1 to 2, 3 to 5, ≥6); and household’s ownership of television (0 = No, 1 = Yes). A pre-calculated wealth quintile, also popularly known as wealth Index, variable is provided in the dataset of NFHS and does not require any manual calculation; it represents the socioeconomic status of the individuals. Further details regarding the generation of the wealth quintile can be accessed from the DHS manual [38].

### Statistical analysis

In the present study, univariate analysis was used to summarize the characteristics of the respondents using weighted percentage and frequencies. To explore the relationship between the exposure and the outcome variables, bivariate analysis was performed using chi-square(χ^2^) tests. The association between the exposure and the outcome variables was analyzed by calculating the adjusted odds ratios (AOR) with 95% confidence interval (CI) using random slope multilevel logistic regression models. The NFHS data is hierarchical in nature, that is, the observations are nested in clusters. So, there are chances that the observations within the clusters may be correlated. If this is the case, a simple logistic regression will underestimate the true variance, leading to an increase in type-1 error [39]. This makes it imperative to control for intra-cluster correlation and cluster-level variations. We achieved this by fitting a two-level random intercept logistic regression model in which clusters were set as level-2 [40]. Since there are four levels in the NFHS-4 data (individual, PSU, district, state), a three-level or four-level regression model could also have been used. But the intra-class correlation for the district or the state levels had a very low value; so the authors decided to use a two-level model and avoid any unnecessary complexity. The benefit of using a multi-level model is that it not only acknowledges the intra-cluster correlation, but also controls the effect of cluster level variables like cluster size, geo-political profile of the cluster, development level of the cluster, accessibility to healthcare facilities in the cluster, etc. The ‘melogit’ command in STATA was utilized to run the models. All the statistical analyses were performed on STATA 16 [41].

## Results

### Background characteristics

Table 1 shows the background characteristics of the participants. It can be seen from the table that the major proportion of the participants was in the age group 25–34 years (56%). The other defining characteristics of the respondents were: residence in a plain area (87%), rural residence (70%), secondary level of educational attainment (47%), belonging to the poorest/poorer (45%) wealth quintile households, practicing Hinduism (79%), belonging to OBC (45%), having a male-headed household (88%), of the 1 to 2 parity (71%), and having a television in the household (56%).

**Table 1:** Background characteristics of women who gave birth in last five years preceding the survey, NFHS-4 (2015–16).

	n	Weighted %
**Age**		
15–24	62,082	34.74
25–34	107,500	55.89
35–49	21,316	9.37
**Ecology of region of residence**^**a**^		
Hilly	25,712	13.47
Plain	165,186	86.53
**Place of Residence**		
Urban	47,833	29.72
Rural	143,065	70.28
**Education**		
No Education	55,165	27.63
Primary	26,712	13.45
Secondary	88,871	46.93
Higher	20,150	12.00
**Wealth Index**		
Poorest	46,782	23.36
Poorer	43,739	21.16
Middle	38,393	19.89
Richer	33,212	19.00
Richest	28,772	16.59
**Religion**		
Hindu	138,343	78.87
Muslim	29,309	16.20
Other	23,246	4.93
**Caste**		
Scheduled Caste	35,170	21.99
Scheduled Tribe	37,889	10.70
OBC	74,060	45.31
Others	34,705	21.13
**Sex of the head of the household**		
Male	167,909	87.81
Female	22,989	12.19
**Parity**		
1 to 2	130,908	71.48
3 to 5	54,195	26.02
≥ 6	5795	2.50
**Household has television**		
No	72,974	35.77
Yes	107,632	56.38

^a^*unweighted*

### Utilization of maternal and child health care services

Table 2 shows the percentage distribution of the MCH services utilization by type of region (hilly/plain) where the respondent usually lives. It can be seen from the table that there is a significant disparity in the utilization of MCH services between hilly and plain regions. The prevalence of skilled birth attendance was 84% in the plain regions, while in the hilly regions, it was only 69%. As many as 61% of the women living in the plain areas received PNC for their last birth; on the other hand, the corresponding figure for women dwelling in the hilly regions was only 50%. There were significant differences in the utilization of ANC, receipt of full immunization, and vaccination against tetanus as well. The percentage of children who did not get any dose of vaccination was 8% in the plains and 15% in the hilly region. In case of IFA consumption, the scenario was the opposite, with the prevalence of ‘IFA consumption for at least 100 days during pregnancy’ being higher in the hilly regions (32%) compared to the plain regions (28%). Fifty-eight percent of the children born in the plain regions received benefits from AWC/ICDS, while this figure was only 50% in the hilly regions. It can also be seen from the table that 35% of the women living in the plain areas and 22% living in the hilly areas received financial assistance from JSY.

**Table 2:** Distribution of utilization of maternal and child healthcare services, NFHS-4 (2015–16)

	Ecology of region of residence		Total
	Hilly	Plain	
Skilled Birth Attendance***	69.05	83.7	81.72
Post-natal care***	49.53	60.8	59.28
Ante-natal care(ns)***	44.90	47.68	47.31
Full Immunization***	43.81	48.60	47.96
No immunization***	14.67	7.77	8.71
At least two tetanus injection before birth***	73.55	83.85	82.48
Given/took iron folic tablet/syrup for at least 100 days during pregnancy***	31.94	27.58	28.16
Child received benefits from Anganwadi/ICDS, last 12 months***	50.01	58.76	57.58
During Pregnancy received benefits from Anganwadi/ICDS***	43.9	57.36	55.54
Received financial assistance for delivery cost***	26.36	38.71	37.05
Financial assistance got from JSY for delivery cost***	21.91	35.24	33.45

*** p-value < 0.001; P-value calculated using chi-square test

### Results of regression analysis

Table 3 displays the results of the multilevel logistic regression analysis, showing the odds ratios of the utilization of MCH services by women dwelling in the hilly regions. The predicted probabilities of the utilization of MCH services by the region of dwelling (hilly/plain) are shown in Table 4. The predicted probabilities were calculated with the help of the *‘margins’ regression post-estimation* command in STATA 16.

**Table 3:** Results of multilevel logistic regression analysis assessing the odds of utilization of maternal and child healthcare services in hilly Himalayan regions of India, NFHS-4 (20015–16)

	Skilled birth attendance	Post-natal care	Ante-natal care	Full Immunization	No Immunization	At least two tetanus injection before birth	Given/took iron folic tablet/syrup for at least 100 days during pregnancy	Child received benefits from Anganwadi/ICDS, last 12 months	During Pregnancy received benefits from Anganwadi/ICDS	Received financial assistance for delivery cost	Financial assistance got from JSY for delivery cost
	**Adjusted Odds Ratio (95% Confidence Interval)**										
**Total**											
Plain	1	1	1	1	1	1	1	1	1	1	1
Hilly	0.42 (0.39–0.45)a	0.56 (0.52–0.60)a	1.08 (1.00–1.17)c	0.82 (0.77–0.86)a	1.62 (1.46–1.78)a	0.46 (0.42–0.49)a	1.74 (1.61–1.88)a	0.96 (0.89–0.1.04)ns	0.74 (0.68–0.82)a	0.54 (0.50–0.58)a	0.43 (0.41–0.47)a
**Urban**											
Plain	1	1	1	1	1	1	1	1	1	1	1
Hilly	0.84 (0.71–0.99)b	0.81 (0.71–0.92)b	1.21 (1.05–1.41)b	0.87 (0.79–0.96)b	1.86 (1.56–2.22)a	0.75 (0.65–0.86)a	1.60 (1.40–1.83)a	1.06 (1.0.90–1.24)ns	0.89 (0.75–1.06)ns	1.05 (0.92–1.21)ns	0.86 (0.75–0.99)c
**Rural**											
Plain	1	1	1	1	1	1	1	1	1	1	1
Hilly	0.34 (0.31–0.37)a	0.48 (0.45–0.52)a	1.06 (0.96–1.17)ns	0.79 (0.74–0.85)a	1.52 (1.34–1.70)a	0.39 (0.36–0.43)a	1.81 (1.65–1.99)a	0.92 (0.84–0.1.01)ns	0.70 (0.63–0.77)a	0.41 (0.38–0.44)a	0.33 (0.31–0.37)a

**a***: p*-value < 0.001; **b**: *p*-value < 0.05; **c**: *p*-value < 0.1; ***ns*** not significant

**Table 4:** Predicted probabilities of utilizing maternal and child healthcare services

	Region	
Variable	Hilly	Plain
Skilled birth attendance	0.72	0.83
Post-natal care	0.50	0.60
Ante-natal care	0.47	0.46
Full Immunization	0.44	0.48
No immunization	0.11	0.08
At least two tetanus injection before birth	0.73	0.84
Given/took iron folic tablet/syrup for at least 100 days during pregnancy	0.36	0.27
Child received benefits from Anganwadi/ICDS, last 12 months	0.58	0.58
During Pregnancy received benefits from Anganwadi/ICDS	0.53	0.56
Received financial assistance for delivery cost	0.27	0.38
Financial assistance got from JSY for delivery cost	0.22	0.35

From Table 3, it can be seen that women living in the hilly areas (AOR: 0.42, 95%CI: 0.39–0.45) had 58% lower odds of receiving SBA than their counterparts in the plain areas. A rural woman living in a hilly region (AOR: 0.42, 95%CI: 0.39–0.45) was 66% less likely to receive SBA than a rural woman living in a plain area. Similarly, women living in the hilly-urban areas (AOR: 0.84, 95%CI: 0.71–0.99) had 16% less odds of receiving SBA than those living in the plain-urban areas. It can also be seen from Table 4 that the predicted probability of receiving SBA was 0.83 for women living in the plain regions, while it decreased to 0.72 for women living in the hilly regions.

Women living in the hilly areas (AOR: 0.56, 95%CI: 0.52–60) were 44% less likely to utilize PNC than those living in the plain regions. In the case of urban areas, women living in the hilly regions (AOR: 0.81, 95%CI: 0.71–92) had 19% less odds of receiving PNC than those living in the plain areas. In the rural areas, women from the hilly regions (AOR: 0.48, 95% CI: 0.45–52) were 52% less likely to receive PNC than their counterparts from the plain regions. The predicted probability of getting PNC was 0.60 for a woman living in a plain region, while for a woman from a hilly region, it reduced to 0.50.

From the results, it can be seen that the odds of making four and more ANC visits were almost similar in both the regions (hilly and plain). However, in the urban areas, women from the hilly regions (AOR: 1.21, 95%CI: 1.05–1.41) had 21% higher odds of receiving for plus ANC than women from the plain areas. It can also be seen from Table 4 that the predicted probabilities of getting ANC were more or less the same in both hilly (0.47) and plain (0.46) regions.

Children born in the hilly regions (AOR: 0.82, 95%CI: 0.77–86) were 18% less likely to receive full immunization than those who born in the plain regions. In both rural and urban areas, children born in the hilly regions were less likely to receive full immunization; however, the odds were much lower in the case of the rural areas. The predicted probabilities of getting full immunization were 0.48 and 0.44 for children born in the plain and the hilly regions respectively. Similarly, the odds (AOR: 1.62, 95%CI: 1.46–1.78) of receiving no immunization were significantly higher among children in the hilly regions compared to their counterparts in the plain regions. The predicted probability of getting no dose of immunization was slightly higher in the hilly regions (0.11) compared with the plain regions (0.08).

The level of vaccination against tetanus among pregnant women was also lower in the hilly regions. A pregnant woman from a hilly area (AOR: 0.46, 95%CI: 0.42–49) was 54% less likely to get ‘at least two injections of tetanus before delivery’ than a woman from a plain region. The odds of receiving tetanus injections were 25% lower for women from the urban-hilly regions (AOR: 0.75, 95%CI: 0.65–86) than women from the urban-plain areas, and 71% lower for women from the rural-hilly areas (AOR: 0.39, 95%CI: 0.36–43) than women from the rural-plain areas. The predicted probability of getting two tetanus injections before birth was significantly higher for women from the plain regions (0.84) compared to those living in the hilly (0.73) regions.

In contrast to everything else, the consumption of IFA tablets/syrup for at least 100 days was higher in the hilly regions. Women from the hilly regions (AOR: 1.74, 95% CI: 1.61–1.88) were 74% more likely to consume IFA tablets/syrup for at least 100 days during pregnancy than those in the plain regions. The predicted probability of consuming IFA during pregnancy was higher among women dwelling in the hilly regions (0.36) compared with those living in the plain regions (0.27).

It can be seen from the table that the odds of a child receiving benefits from AWC/ICDS scheme were not significantly different in the two regions. The predicted probabilities of a child receiving benefits from AWC/ICDS scheme were almost identical for both the regions.

The odds of receiving benefits during pregnancy from AWC/ICDS scheme were 26% lower for women living in the hilly regions (AOR: 0.74, 95%CI: 0.68–0.82) compared to those living in the plain regions. The odds of receiving the benefits were not significantly different for women in the hilly-urban areas and those in the plain-urban areas. In the case of rural areas, women from the hilly regions (AOR: 0.70, 95%CI: 0.63–0.77) had 30% less odds of receiving the benefits than their counterparts from the plain regions. The probability of receiving benefits from AWC/ICDS scheme during pregnancy was 0.56 for women dwelling in the plain regions and 0.53 for those live in the hilly regions.

Women from the hilly regions (AOR: 0.54, 95%CI: 0.50–0.58) had 46% lower odds of receiving financial assistance for covering their delivery cost than women from the plain regions. In the case of rural women also, the odds were lower for the hilly areas (AOR: 0.41, 95%CI: 0.38–0.44) than the plain areas. In the case of urban residence, however, the odds of receiving financial assistance for delivery were not significantly different in the two regions (AOR: 1.05, 95%CI: 0.92–1.21). The predicted probability of receiving financial assistance for covering delivery cost was lower for women living in the hilly regions (0.27) compared with those living in the plain regions (0.38). The utilization of financial assistance under JSY was also significantly lower in the hilly regions. A woman from a hilly region (AOR: 0.43, 95%CI: 0.41–0.47) was 57% less likely to receive financial assistance for delivery under JSY than a woman from a plain region. Similar results were found for rural women. However, among urban women, there was no significant difference between hilly and plain regions in terms of the odds of receiving financial assistance under JSY. The predicted probability for a woman living in a plain region of getting financial assistance under JSY was 0.35, compared with 0.22 for a woman residing in a hilly region.

## Discussion and conclusion

In the present study, we attempted to analyze the level of utilization of maternal and child healthcare services in the hilly Himalayan regions of India and compared it with the rest of the country (plain regions). This study is first of its kind in analyzing the utilization of maternal and child health care services with a focus on the hilly regions of the country. The study found that the utilization of MCH services was significantly lower among the women and children living in the Himalayan states compared with the rest of India. Except for ‘consumption of IFA’ and ‘receipt of benefits from AWC/ICDS by children,’ all other MCH services were significantly underutilized in the hilly regions compared with the plain regions. The study also found that the degree of disparity in the utilization of MCH services between the two regions (hilly and plain) was higher in the rural areas compared to the urban ones.

The Government of India runs various programs to improve maternal and child health in the country. The Child Survival and Safe Motherhood (CSSM) program, launched in 1992, aimed to reduce IMR, provide ANC, and ensure safe delivery. The Reproductive and Child Health (RCH) Programme was launched (Phase-I: 1997 and Phase-II: 2005) to promote institutional delivery and skilled birth attendance and provide emergency obstetric care. The RCH-II programme also focused on minimizing the regional variations in the utilization of MCH and the production of equitable quality healthcare services. The National Rural Health Mission (NRHM) was launched in 2005 with the aim of providing universal access to quality MCH services, especially in rural remote areas, with a special focus on 18 states. Apart from these, several other programs have been launched, such as the Janani Suraksha Yojana (JSY, 2005), the Janani Shishu Suraksha Karyakram (JSSK, 2011). and the National Urban Health Mission (2013). Despite so much emphasis and all the efforts, significant socio-demographic and regional inequalities in the utilization of MCH persist in the country. In this study, we found that the utilization of SBA was significantly lower in the hilly Himalayan region compared to the plain region and that the utilization was particularly alarmingly low in the hilly-rural regions. The utilization of ANC, PNC, and full immunization was also lower in the hilly regions compared to the plain regions. These disparities can be considerably attributed to the difficult landscape of the hilly Himalayan region, resulting in lack of connectivity and poor health infrastructure.

In the previous studies, researchers have tried to map regional variations in the utilization of MCH services. A study conducted in 2012 found that the utilization of maternity care services was lower in the north-eastern, the northern, and the central regions of India compared to the southern region [27, 30]. Other studies have shown that the utilization of MCH services is significantly lower in the rural areas compared to the urban ones [42, 43]. However, so far, no large-scale, comprehensive study using advanced statistical techniques has analyzed the utilization of MCH services while focusing on the topography of the region of residence (hilly/plain). There have been a few small-scale studies in line with the objectives of the present study. For instance, a study conducted in the hilly regions of Uttarakhand analyzed factors affecting the utilization of MCH services. It reported that distance to MCH centers, unavailability of transportation facilities, and absence of doctors at MCH centers were among the main reasons for not availing MCH services in that region [44]. Another study conducted in Meghalaya (a hilly state of India) found that the utilization of at least 4 ANC visits was only to the extent of 11% in the region; however, a major proportion of the study population did receive tetanus vaccination and IFA tablets. The study also found that around 53% of the women lived more than five kilometers away from a healthcare facility, which shows poor availability of healthcare facilities in the region [45].

In India, the population density of the hilly states is low compared with the states in the plain regions [46]. As a result of the scattered distribution of the population and the consequent transportation problems, providing access to healthcare facilities is comparatively challenging in the hilly regions. This underlines the urgent need to identify and address barriers to healthcare utilization in these regions.

From literature and the above discussion, some recommendations can be made to improve MCH services in the hilly regions:

- Promotion of training and appointment of traditional birth attendants (TBA) at the community level: TBAs are a kind of Community Health Workers (CHW), who provide various health and related services, like basic treatment, health education, counselling, and referral activities, to local communities [47]. Previous studies have shown that TBAs can be helpful not only in elevating the utilization of MCH services, but also reducing maternal and child mortality [48].
- Provision of attractive incentives for healthcare workers to working in rural hilly regions: Due to the complex landscape and the unavailability of basic facilities (schools, transport, electricity, etc.), doctors and other healthcare workers usually avoid working in the remote and hilly regions. However, healthcare workers can be drawn to work in these areas by being offered incentives and attractive salaries [49].
- Implementation of a robust connectivity (ambulance) system: As mentioned earlier, distance to the healthcare facility is one of the most important factors associated with the utilization of MCH services. A well-structured ambulance system can help in connecting the remote population with the healthcare facilities.
- Fostering of partnership with community stakeholders: There are always some medical practitioners or leaders at the local level who have a strong connection with the local community and whose instructions people obey. These stakeholders can help in increasing awareness and motivating women to utilize MCH services.
- Promotion of health education and awareness: Due to lack of education and awareness, people in remote areas still believe that pregnancy and childbirth are natural processes that do not require any medical intervention. A mass media based (radio, TV, newspaper, mobile, and internet) program, focusing on increasing awareness regarding the need for and the benefits of MCH services, can be crucial in elevating the utilization of MCH services in the target population.
- Improvement of road connectivity and transportation: One of the major problems in the hilly regions is lack of transportation and poor road connectivity. Many villages in the Himalayan region are still not connected with the national road network due to absence of roads or poor condition of the existing roads. This leads to increase in transportation costs and loss of time, which ultimately results in a lack of motivation to seek MCH services. Development of a robust road network connecting all the nodal points can help in improving the utilization of MCH services in the hilly regions.
- Government of India recently launched Mantri Surakshit Matritva Abhiyan (PMSMA) scheme that aims to provide assured, comprehensive and free-of-cost maternity care to all pregnant women. This study recommends implementation of PMSMA at the lowest level of health facilitate including AWC and ICDS centers, while special attention to hilly regions so that each and every habitat of the region can be covered effectively.

### Limitations

The only limitation of the study is the little bit imprecise categorization of the participants into two categories of region of residence, that is, hilly and plain. In the NFHS data, there is no corresponding or comparable variable. So, in the present study, respondents from the states lying in the Himalayan region were inserted in the ‘hilly’ category and the rest were classified to be living in the ‘plains’ category. There are some hilly areas in the other parts of the country too, like Western Ghats in the southern India, some parts of Andhra Pradesh, and some areas of Madhya Pradesh and Chhattisgarh. However, it was very difficult to identify which of the respondents from these places lived in the hilly areas and which ones lived in the plain areas. Similarly, there are a few plain areas in the Himalayan states as well. But, again, it was difficult to segregate the respondents based on the information available in the NFHS dataset.

## Supplementary Information

**Additional file 1: Supplementary Table-1.** State-wise percentage distribution of utilization of the selected maternal and child healthcare services. **Supplementary Table-2.** Full model results of multilevel logistic regressions assessing adjusted odds ratios of MCH services utilization in various demographic and socioeconomic categories.

## Data Availability

The data utilized for the present study is freely available in public domain through: https://www.dhsprogram.com/data/dataset/India_Standard-DHS_2015.cfm?flag=0.

## Abbreviations

MCH: Maternal and child healthcare
ANC: Ante natal care
PNC: Post natal care
SBA: Skilled birth attendance
ICDS: Integrated child and development services
AOR: Adjusted odds ratio
IFA: Iron folic acid
WHO: World health organization
