# Supplementary material for: Contrast in utilization of maternal and child health services between Himalayan region and rest of India: Evidence from National Family Health Survey (2015–16)
Source: BMC Pregnancy Childbirth. 2021 Sep 5;21:606. doi: 10.1186/s12884-021-04081-0 (PMC8419927; doi:10.1186/s12884-021-04081-0)
Supplement: Supplementary file 1 — Additional file 1: Supplementary Table-1. State-wise percentage distribution of utilization of the selected maternal and child healthcare services. Supplementary Table-2. Full model results of multilevel logistic regressions assessing adjusted odds ratios of MCH services utilization in various demographic and socioeconomic categories. [file 12884_2021_4081_MOESM1_ESM.docx]

| **Supplementary Table-1** | | | |  |  |  |  |  |  |  |  |  |
| --- | --- | --- | --- | --- | --- | --- | --- | --- | --- | --- | --- | --- |
| **State-wise percentage distribution of utilization of selected maternal and child healthcare services** | | | | | | | | | | | | |
|  |  | Maternal and Child Healthcare services | | | | | | | | | | |
|  |  | Skilled birth attendance | Post-natal care | Ante-natal care | Full Immunization | No Immunization | At least two tetanus injection before birth | Given/took iron folic tablet/syrup for at least 100 days during pregnancy | Child received benefits from AWC/ICDS, last 12 months | During Pregnancy received benefits from AWC/ICDS | Received financial assistance for delivery cost | Financial assistance got from JSY for delivery cost |
|  |  | weighted percentage of utilization of the service | | | | | | | | | | |
| **Himalayan range states** | | |  |  |  |  |  |  |  |  |  |  |
|  | Arunachal Pradesh | 56.31 | 26.21 | 25.41 | 27.03 | 24.69 | 55.6 | 9.04 | 20.19 | 14.49 | 13.99 | 10.52 |
|  | Himachal Pradesh | 80.22 | 68.41 | 67.44 | 54.94 | 5.23 | 69.85 | 50.24 | 75.5 | 78.87 | 17.1 | 11.31 |
|  | Jammu & Kashmir | 86.76 | 71.61 | 80.77 | 60.87 | 5.08 | 81.5 | 28.48 | 35.89 | 28.72 | 51.98 | 47.44 |
|  | Meghalaya | 58.78 | 49.37 | 51.89 | 48.56 | 12.81 | 69.23 | 39.54 | 53.2 | 53.3 | 18.71 | 15.2 |
|  | Mizoram | 82.97 | 61.07 | 56.77 | 43.63 | 25.94 | 74.64 | 54.31 | 71.65 | 74.4 | 41.71 | 38.06 |
|  | Nagaland | 42.88 | 21.14 | 13.51 | 27.77 | 21.16 | 60.23 | 4.24 | 38.37 | 10.63 | 20.05 | 11.9 |
|  | Sikkim | 97.78 | 74.53 | 77.98 | 65.75 | 3.08 | 95.88 | 55.87 | 54.04 | 51.95 | 33.48 | 30.7 |
|  | Uttarakhand | 75.33 | 54.44 | 30.06 | 48.66 | 6.64 | 86.81 | 26.6 | 68.21 | 58.12 | 39.52 | 37.26 |
| **Plain region states** | | | | | |  |  |  |  |  |  |  |
|  | Andhra Pradesh | 95.9 | 78.55 | 75.79 | 44.55 | 4.38 | 92.05 | 57.57 | 75.16 | 82.46 | 20.04 | 16.11 |
|  | Assam | 75.38 | 54.7 | 46.1 | 43.55 | 14.16 | 84.66 | 33.51 | 59.41 | 60.61 | 52.73 | 50.53 |
|  | Bihar | 74.73 | 44.74 | 14.66 | 47.26 | 8.02 | 83.04 | 9.78 | 55.44 | 41.73 | 40.87 | 38.94 |
|  | Chhattisgarh | 79.24 | 61.63 | 57.01 | 54.27 | 2.4 | 90.04 | 30.21 | 81.86 | 87.6 | 49.14 | 48.23 |
|  | Goa | 99.14 | 91.64 | 88.12 | 70.81 | 1.45 | 87.9 | 63.19 | 63.87 | 69.16 | 15.85 | 6.92 |
|  | Gujarat | 90.08 | 60.63 | 67.23 | 39.84 | 11.3 | 82.07 | 36.89 | 67.05 | 64.02 | 13.49 | 10.35 |
|  | Haryana | 88.77 | 66.11 | 45.66 | 50.48 | 7.94 | 85.84 | 33.15 | 48.75 | 41.6 | 12.86 | 11.25 |
|  | Jharkhand | 71.83 | 43.58 | 29.97 | 51.27 | 5.3 | 86.39 | 15.65 | 64.39 | 71.63 | 28.3 | 27.68 |
|  | Karnataka | 97.42 | 62.49 | 74.25 | 50.4 | 6.06 | 81.5 | 43.49 | 67.92 | 68.38 | 28.12 | 22.27 |
|  | Kerala | 99.95 | 88.25 | 96.58 | 71.16 | 1.42 | 95.71 | 75.56 | 53.73 | 32.58 | 23.74 | 20.69 |
|  | Madhya Pradesh | 83.54 | 54.94 | 35.52 | 40.64 | 7.92 | 83.38 | 23.68 | 69.32 | 72.68 | 52.99 | 49.99 |
|  | Maharashtra | 93.95 | 76 | 72.8 | 47.74 | 9.05 | 82.46 | 40.11 | 55.74 | 50.75 | 10.49 | 8.41 |
|  | Manipur | 76.63 | 60.83 | 65.34 | 51.25 | 8.47 | 84.62 | 36.81 | 32.78 | 27.55 | 24.77 | 18.98 |
|  | Delhi | 92.75 | 62.08 | 72.07 | 57.85 | 3.66 | 84.13 | 51.1 | 15.07 | 10.52 | 7.18 | 5.74 |
|  | Odisha | 89.26 | 73 | 65.36 | 61.53 | 7.14 | 90.97 | 39.43 | 84.49 | 89.81 | 68.58 | 63.99 |
|  | Punjab | 96.39 | 87.2 | 68.19 | 73.71 | 1.49 | 89.35 | 43.02 | 64.46 | 63.55 | 25.68 | 19.72 |
|  | Rajasthan | 88.94 | 64.92 | 38.52 | 43.16 | 8.64 | 82.45 | 16.68 | 43.97 | 49.7 | 54.14 | 49.09 |
|  | Tamil Nadu | 99.63 | 73.76 | 81.54 | 56.73 | 3.9 | 64.73 | 63.72 | 64.8 | 64.65 | 54.98 | 30.3 |
|  | Tripura | 81.59 | 61.42 | 66.43 | 47.41 | 15.58 | 90.99 | 14.21 | 62.66 | 56.03 | 29.71 | 28.6 |
|  | Uttar Pradesh | 74.28 | 54.34 | 27.45 | 38.67 | 10.84 | 82.03 | 12.39 | 44.95 | 42.02 | 34.47 | 33.81 |
|  | West Bengal | 84.04 | 62.12 | 75.12 | 67.04 | 2.83 | 90.39 | 28.11 | 75.29 | 72.87 | 26.2 | 23.59 |
|  | Telangana | 93.98 | 80.73 | 73.31 | 48.87 | 3.8 | 86.29 | 50.89 | 68.91 | 69.46 | 15.91 | 12.36 |

| **Supplementary Table-2** | | |  |  |  |  |  |  |  |  |  |
| --- | --- | --- | --- | --- | --- | --- | --- | --- | --- | --- | --- |
| **Results of multilevel logistic regressions assessing adjusted odds ratios of MCH services utilization in various demographic and socioeconomic categories** | | | | | | | | | | | |
|  | **Adj. Odds Ratio** | | | | | | | | | | |
|  | **(95% CI)** | | | | | | | | | | |
|  | Skilled birth attendance | Post-natal care | Ante-natal care | Full Immunization | Received zero dose of immunization | At least two tetanus injection before birth | Given/took iron folic tablet/syrup for at least 100 days during pregnancy | Child received benefits from Anganwadi/ICDS last 12 months | During Pregnancy received benefits from Anganwadi/ICDS | Received financial assistance for delivery cost | Financial assistance got from JSY for delivery cost |
| **Region** |  |  |  |  |  |  |  |  |  |  |  |
| Plain® |  |  |  |  |  |  |  |  |  |  |  |
| Hilly | 0.42*** | 0.56*** | 1.08* | 0.82*** | 1.62*** | 0.46*** | 1.74*** | 0.96ns | 0.74*** | 0.54*** | 0.44*** |
|  | (0.39 - 0.45) | (0.52 - 0.60) | (1.00 - 1.17) | (0.77 - 0.86) | (1.46 - 1.78) | (0.42 - 0.49) | (1.61 - 1.88) | (0.89 - 1.04) | (0.68 - 82) | (0.50 - 0.57) | (0.40 - 0.47) |
| **Age of the woman** |  |  |  |  |  |  |  |  |  |  |  |
| 15 - 24® |  |  |  |  |  |  |  |  |  |  |  |
| 25-34 | 1.07 ns | 1.09*** | 1.09*** | 1.71*** | 0.87*** | 1.07*** | 1.13*** | 0.88*** | 0.93*** | 1.05*** | 1.06*** |
|  | (0.98 - 1.16) | (1.05 - 1.12) | (1.06 - 1.27) | (1.67 - 1.76) | (0.82 - 0.91) | (1.03 - 1.11) | (1.09 - 1.67) | (0.85 - 0.91) | (0.90 - 0.96) | (1.03 - 1.09) | (1.03 - 1.09) |
| 35-49 | 0.97 ns | 1.16*** | 1.14*** | 2.23*** | 0.88*** | 1.04ns | 1.21*** | 0.74*** | 0.78*** | 0.91*** | 0.92*** |
|  | (0.85 - 1.11) | (1.09 - 1.23) | (1.08 - 1.21) | (2.13 - 2.33) | (0.82 - 0.95) | (0.98 - 1.11) | (1.14 - 1.29) | (0.70 - 0.78) | (0.74 - 0.82) | (0.87 - 0.96) | (0.87 - 0.97)** |
| **Place of residence** |  |  |  |  |  |  |  |  |  |  |  |
| Urban® |  |  |  |  |  |  |  |  |  |  |  |
| Rural | 0.75*** | 0.99ns | 0.77*** | 1.05*** | 0.94* | 0.98ns | 0.91*** | 2.70*** | 3.26*** | 1.33*** | 1.37*** |
|  | (0.67 - 0.84) | (0.94 - 1.05) | (0.73 - 0.81) | (1.01 - 1.09) | (0.87 - 1.01) | (0.93 - 1.03) | (0.87 - 0.96) | (2.56 - 2.84) | (3.07 - 3.45) | (1.26 - 1.39) | (1.30 - 1.40) |
| **Wealth Index** |  |  |  |  |  |  |  |  |  |  |  |
| Poorest® |  |  |  |  |  |  |  |  |  |  |  |
| Poorer | 1.18*** | 1.06** | 1.39*** | 1.07*** | 0.88*** | 1.07*** | 1.19*** | 0.99ns | 0.97ns | 1.08** | 1.06** |
|  | (1.07 - 1.29) | (1.01 - 1.11) | (1.33 - 1.45) | (1.04 - 1.12) | (0.83 - 0.93) | (1.02 - 1.12) | (1.13 - 1.25) | (0.95 - 1.03) | (0.93 - 1.01) | (1.04 - 1.25) | (1.02 - 1.10) |
| Middle | 1.62*** | 1.20*** | 1.71*** | 1.18*** | 0.81*** | 1.10*** | 1.39*** | 0.89*** | 0.86*** | 0.94** | 0.90** |
|  | (1.44 - 1.81) | (1.14 - 1.27) | (1.63 - 1.81) | (1.13 - 1.23) | (0.76 - 0.88) | (1.04 - 1.16) | (1.31 - 1.47) | (0.85 - 0.93) | (0.82 - 0.90) | (0.90 - 0.0.99) | (0.85 - 0.0.94) |
| Richer | 2.19*** | 1.37*** | 2.13*** | 1.24*** | 0.73*** | 1.13*** | 1.70*** | 0.74*** | 0.71*** | 0.69*** | 0.65*** |
|  | (1.89 - 2.52) | (1.29 - 1.46) | (2.01 - 2.26) | (1.19 - 1.30) | (0.67 - 0.80) | (1.06 - 1.20) | (1.59 - 1.82) | (0.70 - 0.78) | (0.67 - 0.75) | (0.66 - 0.0.73) | (0.62 - 0.69) |
| Richest | 2.74*** | 1.55*** | 3.01*** | 1.35*** | 0.63*** | 1.29*** | 2.38*** | 0.53*** | 0.46*** | 0.38*** | 0.36*** |
|  | (2.25 - 3.34) | (1.44 - 1.67) | (2.81 - 3.22) | (1.28 - 1.43) | 0.56 - 0.71) | (1.19 - 1.39) | (2.21 - 2.57) | (0.50 - 0.57) | (0.43 - 0.49) | (0.35 - 0.40) | (0.34 - 0.39) |
| **Education level** |  |  |  |  |  |  |  |  |  |  |  |
| No education® |  |  |  |  |  |  |  |  |  |  |  |
| Primary | 1.10ns | 1.08*** | 1.25*** | 1.20*** | 0.76*** | 1.19*** | 1.05* | 1.15*** | 1.14*** | 1.15*** | 1.16*** |
|  | (0.99 - 1.20) | (1.03 - 1.13) | (1.95 - 1.31) | (1.16 - 1.24) | (0.72 - 0.81) | (1.13 - 1.25) | (1.00 - 1.11) | (1.11 - 1.20) | (1.09 - 1.19) | (1.11 - 1.20) | (1.12 - 1.21) |
| Secondary | 1.43*** | 1.13*** | 1.52*** | 1.28*** | 0.67*** | 1.31*** | 1.24*** | 1.11*** | 1.10*** | 1.25*** | 1.24*** |
|  | (1.31 - 1.55) | (1.08 - 1.18) | (1.46 - 1.58) | (1.24 - 1.33) | (0.63 - 0.71) | (1.26 - 1.37) | (1.18 - 1.30) | (1.07 - 1.16) | (1.06 - 1.48) | (1.21 - 1.30) | (1.20 - 1.29) |
| Higher | 1.74*** | 1.27*** | 2.03*** | 1.17*** | 0.62*** | 1.43*** | 1.71*** | 0.79*** | 0.70*** | 0.87*** | 0.87*** |
|  | (1.40 - 2.15) | (1.19 - 1.35) | (1.91 - 2.15) | (1.12 - 1.23) | 0.59 - 0.69) | (1.33 - 1.54) | (1.61 - 1.83) | (0.74 - 0.83) | (0.66 - 0.75) | (0.83 - 0.93) | (0.82 - 0.92) |
| **Religion** |  |  |  |  |  |  |  |  |  |  |  |
| Hindu® |  |  |  |  |  |  |  |  |  |  |  |
| Muslim | 0.90* | 1.02ns | 1.17*** | 0.80*** | 1.59*** | 0.88*** | 0.94* | 0.80*** | 0.71*** | 0.76*** | 0.76*** |
|  | 0.80 - 1.01 | (0.96 - 1.08) | (1.11 - 1.24) | (0.76 - 0.83) | (1.48 - 1.71) | (0.84 - 0.93) | (0.89 - 1.00) | (0.76 - 0.83) | (0.67 - 0.75) | (0.72 - 0.80) | (0.72 - 0.80) |
| Christian | 1.08ns | 0.93* | 0.74*** | 0.81*** | 1.91*** | 0.85*** | 0.93ns | 0.60*** | 0.49*** | 0.78*** | 0.78*** |
|  | (0.89 - 1.31) | (0.85 - 1.01) | (0.68 - 0.81) | (0.75 - 0.86) | (1.71 - 2.14) | (0.78 - 0.93) | (0.85 - 1.02) | (0.55 - 0.65) | (0.45 - 0.54) | (0.72 - 0.85) | (0.71 - 0.85) |
| Sikh | 3.58*** | 1.87*** | 2.13*** | 2.03*** | 0.39*** | 1.08ns | 1.91*** | 2.08*** | 1.94*** | 0.58*** | 0.46*** |
|  | (2.32 - 5.52) | (1.58 - 2.20) | (1.84 - 2.47) | (1.84 - 2.24) | (0.28 - 0.55) | (0.92 - 1.27) | (1.68 - 2.18) | (1.83 - 2.36) | (1.69 - 2.23) | (0.52 - 0.65) | (0.40 - 0.52) |
| Other | 0.99ns | 0.92ns | 1.10* | 1.01ns | 1.19** | 0.87** | 0.90* | 0.62*** | 0.60*** | 0.64*** | 0.65*** |
|  | (0.79 - 1.26) | (0.82 - 1.03) | (0.98 - 1.23) | (0.93 -1.10) | (1.03 - 1.38) | (0.78 - 0.97) | (0.80 - 1.00) | (0.55 - 0.68) | (0.54 - 0.67) | (0.58 - 0.71) | (0.58 - 0.71) |
| **Caste** |  |  |  |  |  |  |  |  |  |  |  |
| General® |  |  |  |  |  |  |  |  |  |  |  |
| SC | 0.88* | 0.93*** | 0.84*** | 0.98ns | 0.90** | 0.95* | 0.87*** | 1.41*** | 1.65*** | 1.43*** | 1.37*** |
|  | (0.77 - 1.01) | (0.88 - 0.98) | (0.80 - 0.88) | (0.93 - 1.013) | (0.83 - 0.98) | (0.89 - 0.99) | (0.82 - 0.92) | (1.34 - 1.47) | (1.57 - 1.73) | (1.37 - 1.50) | (1.31 - 1.44) |
| ST | 0.71*** | 0.96ns | 0.88*** | 0.90*** | 1.06ns | 0.91*** | 0.90*** | 1.36*** | 1.62*** | 1.29*** | 1.31*** |
|  | (0.61 - 0.82) | (0.90 - 1.03) | (0.83 - 0.94) | (0.86 - 0.94) | (0.97 - 1.16) | (0.85 - 0.97) | (0.85 - 0.96) | (1.28 - 1.44) | (1.52 - 1.72) | (1.22 - 1.37) | (1.23 - 1.38) |
| OBC | 0.97ns | 0.89*** | 0.77*** | 0.99ns | 0.93** | 0.99ns | 0.87*** | 1.16*** | 1.32*** | 1.29*** | 1.28*** |
|  | (0.86 - 1.08) | (0.85 - 0.93) | (0.74 - 0.81) | (0.95 - 1.02) | (0.86 - 0.99) | (0.94 - 1.04) | (0.83 - 0.91) | (1.12 - 1.21) | (1.26 - 1.37) | (1.24 - 1.35) | (1.23 - 1.34) |
| **Sex of the head of the household** |  |  |  |  |  |  |  |  |  |  |  |
| Male® |  |  |  |  |  |  |  |  |  |  |  |
| Female | 1.05ns | 0.98ns | 0.96* | 1.03* | 0.90*** | 1.01ns | 1.02ns | 1.00ns | 0.99ns | 1.01ns | 0.99ns |
|  | (0.94 - 1.16) | (0.94 - 1.03) | (0.92 - 1.00) | (1.00 - 1.07) | (0.85 - 0.97) | (0.96 - 1.58) | (0.98 - 1.07) | (0.96 - 1.04) | (0.95 - 1.03) | (0.97 - 1.05) | (0.95 - 1.03) |
| **Child Marriage** |  |  |  |  |  |  |  |  |  |  |  |
| No® |  |  |  |  |  |  |  |  |  |  |  |
| Yes | 0.94* | 0.91*** | 0.88*** | 1.14*** | 1.03ns | 0.99ns | 0.90*** | 0.92*** | 0.92*** | 0.96** | 0.97** |
|  | (0.88 - 1.01) | (0.89 - 0.95) | (0.86 - 0.91) | (1.12 - 1.74) | (0.98 - 1.07) | (0.96 - 1.02) | (0.87 - 0.93) | (0.90 - 0.95) | (0.89 - 0.94) | (0.93 - 0.99) | (0.94 - 0.99) |
| **Parity** |  |  |  |  |  |  |  |  |  |  |  |
| 1-2® |  |  |  |  |  |  |  |  |  |  |  |
| 3-5 | 1.07ns | 0.87*** | 0.70*** | 0.75*** | 1.14*** | 0.81*** | 0.80*** | 1.09*** | 1.10*** | 0.78*** | 0.81*** |
|  | (0.98 - 1.16) | (0.84 - 0.90) | (0.67 - 0.72) | (0.73 - 0.77) | (1.08 - 1.20) | (0.78 - 0.84) | (0.77 - 0.83) | (1.05 - 1.12) | (1.06 1.14) | (0.76 - 0.81) | (0.79 - 0.84) |
| 5+ | 0.97ns | 0.71*** | 0.48*** | 0.56*** | 1.56*** | 0.56*** | 0.73*** | 1.10*** | 1.05ns | 0.71*** | 0.71*** |
|  | (0.85 - 1.11) | (0.64 - 0.79) | (0.43 - 0.53) | (0.52 - 0.60) | (1.41 - 1.73) | (0.51 - 0.61) | (0.64 - 0.82) | (1.02 - 1.19) | (0.97 - 1.14) | (0.61 - 0.77) | (0.69 - 0.82) |
| **Type of delivery** |  |  |  |  |  |  |  |  |  |  |  |
| Home® |  |  |  |  |  |  |  |  |  |  |  |
| Institutional | - | 75.27 | 2.53*** | 1.36*** | 0.39*** | 1.99*** | 1.89*** | 1.28*** | 1.39*** | - | - |
|  | - | (70.48 - 80.38) | (2.43 - 2.63) | (1.32 - 1.40) | (0.37 - 0.41) | (1.92 - 2.07) | (1.80 - 1.97) | (1.24 - 1.33) | (1.34 - 1.44) | - | - |
| **Frequency of watching television** |  |  |  |  |  |  |  |  |  |  |  |
| not at all® |  |  |  |  |  |  |  |  |  |  |  |
| less than once a week | 1.21*** | 1.10*** | 1.23*** | 1.07*** | 0.82*** | 1.16*** | 0.94ns | 1.22*** | 1.26*** | 1.13*** | 1.13*** |
|  | (1.08 - 1.36) | (1.04 - 1.17) | (1.17 - 1.31) | (1.02 - 1.12) | (0.76 - 0.89) | (1.09 - 1.23) | (0.88 - 1.01) | (1.56 - 1.28) | (1.19 - 1.33) | (1.07 - 1.87) | (1.07 - 1.88) |
| at least once a week | 1.34*** | 1.18*** | 1.30*** | 1.10*** | 0.84*** | 1.20*** | 1.03ns | 1.22*** | 1.28*** | 1.17*** | 1.16*** |
|  | (1.20 - 1.51) | (1.11 - 1.25) | (1.23 - 1.37) | (1.05 - 1.15) | (0.78 - 0.91) | (1.13 - 1.27) | (0.97 - 1.09) | (1.16 - 1.28) | (1.22 - 1.35) | (1.12 - 1.23) | (1.10 - 1.22) |
| almost every day | 1.42*** | 1.41*** | 1.66*** | 1.25*** | 0.74*** | 1.28*** | 1.20*** | 1.36*** | 1.42*** | 1.22*** | 1.21*** |
|  | (1.29 - 1.57) | (1.34 - 1.47) | (1.59 - 1.74) | (1.21 - 1.29) | (0.70 - 0.79) | (1.22 - 1.34) | (1.15 - 1.27) | (1.31 - 1.42) | (1.36 - 1.48) | 1.17 - 1.27) | 1.17 - 1.27) |
|  |  |  |  |  |  |  |  |  |  |  |  |
| ICC(standard error) | 0.35(0.012) | 0.35(0.004) | 0.38(0.004) | 0.16(0.003) | 0.35(0.006) | 0.28(0.005) | 0.33(0.005) | 0.34(0.004) | 0.43(0.004) | 0.34(0.004) | 0.34(0.004) |
|  | (0.32 - 0.37) | (0.33 - 0.37) | (0.37 - 0.39) | (0.15 - 0.17) | (0.33 - 0.36) | (0.26 - 0.27) | (0.32 - 0.34) | (0.33 - 0.35) | (0.42 - 0.44) | (0.33 - 0.35) | (0.33 - 0.35) |
| ***Note: *******: p-value < 0.001;* *********: p-value < 0.05;* ********: p-value <0.1;* ***ns****: not significant* | | | | | | | | | | | |
